# 2221. Use of PCR and MLST analysis to establish *Treponema pallidum* as the cause of a painful oral lesion

**DOI:** 10.1093/ofid/ofac492.1840

**Published:** 2022-12-15

**Authors:** Maria Rosa Velasquez Espiritu, Gary P Wormser, Kaitlin Cafferky, Diane Edmondson, Steven Norris, Eric Munzer, Ciril-Christian Rizk, Irina Gelman, Jacqueline Lawler, Heather Boss, Marina Keller

**Affiliations:** New York Medical College - Metropolitan Hospital / Westchester Medical Center, New York, New York; Westchester Medical Center, New York, New York; Orange County Department of Health, Middletown, New York; McGovern Medical School, UT Health, Houston, Texas; McGovern Medical School, UT Health, Houston, Texas; ENT and Allergy Associates, New Windsor, New York; CBL Path Laboratory, Rye Brook, New York; Orange County Department of Health, Middletown, New York; Orange County Department of Health, Middletown, New York; Orange County Department of Health, Middletown, New York; Westchester Medical Center, New York, New York

## Abstract

**Background:**

A syphilitic chancre is described classically as a single, indurated, and painless ulcer at the site of *Treponema pallidum* inoculation.

**Methods:**

We present a 39-year-old heterosexual, monogamous, HIV-negative woman with a painful tongue lesion of one month’s duration. To exclude a malignancy, a biopsy was performed.

**Results:**

Histopathology showed intense inflammatory reaction, and *T. pallidum* immunocytochemistry demonstrated a high density of spirochetes in the subepithelial connective tissue. Subsequent testing revealed a reactive anti-*T. pallidum* IgG EIA and an RPR titer of 1:8. PCR amplification and multi-locus sequence typing (MLST) confirmed that the visualized spirochetes were *T. pallidum*. This particular MLST strain pattern has been identified twice before and named ST-109; however, no clinical information is available.

Diagnosis and appropriate treatment were delayed since syphilis was not suspected initially. When the results became available, the patient was treated with 2.4 million units of benzathine penicillin given IM with complete resolution of the lesion. The male partner, who reported no symptoms, had a positive *T. pallidum* EIA with an RPR titer of 1:64 and was also treated with penicillin. Within four months, a woman from an adjacent town presented to urgent care with three perineal severely painful lesions, testing negative for HIV, *Herpes Simplex Virus* (HSV) by PCR of an ulcer swab, and chlamydia and gonorrhea on a urine sample by a NAAT. The anti-*T. pallidum* EIA was reactive, and the RPR titer was 1:4. The lesions resolved with IM penicillin treatment.

**Conclusion:**

The observations reported here indicate that a subset of early syphilis cases may present with painful lesions. Despite the commonly held belief that syphilitic chancres are painless, several case series have reported tender lesions, single or multiple, without evidence of concurrent HSV infection to explain the pain. This is the first PCR and MLST sequencing analysis of a *T. pallidum* strain within a painful chancre. Given the rise in the number of cases of syphilis in the USA, it is essential that clinicians be aware of this atypical presentation. It remains to be determined whether such painful lesions are only associated with certain *T. pallidum* genotypes.

**Disclosures:**

**All Authors**: No reported disclosures.

